# Reliability and Validity of the Chinese Nine-Item Basic Psychological Need Satisfaction Scale Based on Self-Determination Theory With Older Adults

**DOI:** 10.3389/fpsyg.2021.751631

**Published:** 2021-12-08

**Authors:** Yuting Yang, Miao Yao, Yongwei Yang, Qiong Ye, Ting Lin

**Affiliations:** School of Nursing, Fujian Medical University, Fuzhou, China

**Keywords:** self-determination theory, Basic Psychological Need Satisfaction Scale, older adults, psychological assessment, validity

## Abstract

**Background:** Self-determination theory distinguishes three basic human psychological needs: competence, relatedness, and autonomy. The measurement of these needs in populations of older adults has been limited and inadequate. Yet, results from such an assessment are likely to be valuable in policymaking, specifically toward the goal of healthy aging.

**Aim:** The objective of this study was to test the reliability and validity of the Chinese version of the Basic Psychological Need Satisfaction Scale (BPNS) based on self-determination theory with older adults.

**Methods:** A total of 809 older adults were invited to participate in this study. We examined the item analysis, internal reliability, factorial validity, criterion validity, and measurement invariance across sex of a Chinese translation of the BPNS.

**Results:** The findings demonstrated that the scale had a good factorial validity, criterion validity, and satisfactory internal reliability. All the items were qualified according to item analysis (*p* < 0.001). The Cronbach’s α coefficient for the total scale was 0.877. The coefficients of three subscales were 0.826 (autonomy), 0.807 (competence), and 0.847 (relatedness). Exploratory factor analysis indicated three factors that explained 75.12% of the total variance. Confirmatory factor analysis showed measurement fit exceeded the recommended criteria in all the cases. Measurement invariance analysis manifested that the factor loadings, factor variances and covariances, and residuals to measurement structure were invariant across the male and female participants.

**Conclusion:** The Chinese version of the BPNS based on self-determination theory was proven to be reliable and valid. The usability of the scale to assess the satisfaction of the basic psychological needs of older adults in China was demonstrated.

## Introduction

In the 1980s, American psychologists Deci and Ryan (1980) proposed self-determination theory (SDT). Inductively and based on extensive empirical studies, it distinguished three basic human psychological needs: competence, relatedness, and autonomy (Ryan and Deci, 2000). Autonomy is a form of functioning associated with feelings of volition, coherence, and integration. The performance of autonomy is when one’s behavior is self-endorsed or congruent with one’s true interests and values (Ryan and Deci, 2017). Competence refers to the basic needs to feel efficient and to feel mastery. People could operate effectively within their crucial life contexts (Ryan and Deci, 2017). Relatedness is experiencing oneself as giving or contributing to others. Both feel connecting to close friends and family members and be significant members of social groups, humans experience relatedness, and belonging (Ryan and Deci, 2017). The satisfaction of each of the three basic psychological needs is related. Satisfied demands induce intrinsic motivation and potential, promote health and development, and enhance happiness.

Scholars have compiled and revised relevant scales to measure these three basic psychological needs. For example, Gagné (2003) developed the 21-item Basic Psychological Need Satisfaction Scale (BPNS). Sheldon et al. (2001) compiled the 30-item scale to measure 10 kinds of human psychological needs. Afterward, Sheldon and Niemiec (2006) concluded the 9-item scale from the 30-item scale (Sheldon et al., 2001) and tested 315 American college students three basic psychological needs by an online survey. The reliability for each subscale was as follows: autonomy (0.76), competence (0.84), and relatedness (0.88). Besides, subjective well-being (SWB) and happiness could be used as good criteria for the three basic psychological needs when measuring criterion validity. Above studies verified that the scale has good reliability and validity for that population and proved the relationship between the various needs assessed and happiness.

Chinese researchers have introduced and revised different versions of the SDT scale. Dongjie et al. (2012) translated Gagné’s scale (Gagné, 2003) to simplified Chinese and recruited 6,366 young workers with stratified sampling in five state-owned enterprises in Chinese eastern, central, and western regions. The revised scale consisted of 15 items with four factors – satisfied state of autonomy need, blocked state of autonomy need, satisfied state of relatedness need, and blocked state of relatedness need. It showed that the construct of the Chinese version of the scale (Gagné, 2003) had certain flaws and it was impossible to measure Chinese competence needs of young workers. Junsheng et al. (2013) translated the scale (Gagné, 2003) again to simplified Chinese and investigated 526 elementary and middle school students in Shanghai. The revised scale was the 19-item version. The scale showed good construct validity, the total Cronbach’s α coefficient, and test-retest reliability, while the three subscales Cronbach’s α coefficient were 0.57 for autonomy, 0.62 for competence, and 0.72 for relatedness, showing an insufficient Cronbach’s α coefficient of the subscale. Later, Jian et al. (2020) translated the 9-item BPNS (Sheldon and Niemiec, 2006) to simplified Chinese, surveying it with 291 Chinese college students on the spot in 2020. The results showed that each item was qualified and should be maintained. Exploratory factor analysis (EFA) showed the scale consisted of three factors, accounting for 64.73% of the total variance. Confirmatory factor analysis (CFA) revealed three factors model fit well [chi-squared (χ^2^) (24) = 40.360, degrees of the freedom (χ^2^/*df*) = 1.68, goodness of fit index (GFI) = 0.940, comparative fit index (CFI) = 0.970, normed fit index (NFI) = 0.940, incremental fit index (IFI) = 0.970, and root mean square error of approximation (RMSEA) = 0.070]. The scores of three subscales positively correlated with life satisfaction, positive affect, affect balance, and subjective well-being, while negatively correlated with negative affect. The total Cronbach’s α coefficient is 0.86 and three subscales are 0.82 (autonomy), 0.86 (competence), and 0.80 (relatedness). It indicated that the scale is sufficiently homogeneous and item discrimination and presented good reliability, construct validity, and criterion validity used for Chinese college students.

None of mentioned above Chinese versions of the scales has been applied in older adults as subjects. As it is not yet clear if existing questionnaires are suitable for measuring satisfaction of the older adults with their needs, it is valuable to test their reliability and validity with that population. This study selected the Chinese version of the BPNS revised by Jian et al. (2020) as its tool, because of the characteristics of the elderly, to discover an appropriate scale to measure the basic psychological needs of older adults.

## Materials and Methods

### Participants and Procedures

A total of 809 convenience sample was recruited from parks, communities, and wards of a tertiary hospital in Fuzhou, China, between October 2020 and May 2021. The inclusion criteria included age older than 60-year-old, living in an urban community 12 months or more, clear cognition, ability to express and comprehend simple Chinese characters, and willingness to complete questionnaires. The exclusion criteria were hearing impairment, speech impairment, cognitive impairment, mental illness, and severe or terminal ill.

It is generally accepted that a minimum sample size of 300 is required for EFA (Yong and Pearce, 2013), while a sample size of 300 is also considered adequate for CFA (MacCallum et al., 1999). What is more, two independent samples were recommended to be used to perform EFA and CFA when analyzing the psychometric properties, which were considered more effective than using one sample to analyze both (Woods and Edwards, 2007). The half sample (404 participants) was randomly selected to participate in the EFA and the remaining half sample (405 participants) was randomly participated in the CFA by the Research Randomizer website^1^.

### Measures

#### Chinese Version of the Basic Psychological Need Satisfaction Scale

The BPNS (Sheldon and Niemiec, 2006) translated into simplified Chinese (Jian et al., 2020) was used in this study. The Chinese version of the BPNS comprises 9-item grouped into three subscales, with 3-item each for assessing one of the needs, included autonomy, competence, and relatedness. Responses are provided on a 7-point Likert scale ranging from 1 (strongly disagree) to 7 (strongly agree). The average score of 3-item contained in each dimension has expressed each need satisfaction. The average score of 9-item has expressed the total need satisfaction. The higher the score, the higher the satisfaction. The total Cronbach’s α coefficient is 0.86 and three subscales are 0.82 (autonomy), 0.86 (competence), and 0.80 (relatedness), indicating good reliability and validity in Chinese college students (Jian et al., 2020). In this study, 809 participants completed the Chinese version scale.

#### Multiple Happiness Questionnaire (MHQ)

Satisfying the three basic psychological needs could increase happiness (Ryan and Deci, 2000). The MHQ (Yuanjiang, 2003) is a 50-item simplified Chinese scale with nine domains including life satisfaction (5 items), positive affect (6 items), negative affect (6 items), subjective vitality (6 items), self-worth (5 items), health concern (5 items), positive relation (3 items), altruism commitment (5 items), and personal growth (9 items). Item 12 and item 14 are reverse scored. The happiness index only has a nine-point question. A higher score indicates better happiness. The total Cronbach’s α coefficient is 0.909 in old adults (Yuanjiang and Hongying, 2008). The internal consistency coefficient is 0.692–0.912, split-half reliability is 0.645–0.911, and the scale is appropriate for the Chinese elderly. This study compared the similarity of three dimensions and total scores of the Chinese version of the BPNS (Jian et al., 2020) and dimensions scores of the MHQ to test criterion validity. Because the MHQ has many questions, some participants were unwilling to take a long time to finish it during COVID-19 pandemic. Finally, only 507 participants answered the MHQ from 809 eligible ones integrally (the MHQ response rate of 62.67%) when necessary to complete two scales simultaneously.

### Data Collection

Before the collection stage, the Ethical Review Committee of Fujian Medical University approved this study. The investigators consisted of three graduate students and several undergraduates in Fujian Medical University. In the preparation stage, the investigators had been uniformly trained, emphasized the application of uniform instructions, and underwent one or two preinvestigation trainings. Before the formal investigation, the investigators informed participants of the study purpose, significance, usage, privacy rights, etc., signed the informed consent, and then conducted one-to-one questionnaire surveys. If participants were illiterate or did not understand the content, investigators explained on the spot and asked for permission to fill out the questionnaire. After finishing the questionnaire, investigators immediately checked it. If the questionnaires were missing items or had obvious logical errors, the participants filled in or modified them on the spot. After checking again, the investigators withdraw the questionnaire. Finally, participants were usually able to fill in the Chinese version of the BPNS (Jian et al., 2020) in 5–10 min and the MHQ (Yuanjiang, 2003) in 20–30 min.

After collecting data, researchers used rigorous numbers instead of names to mark the questionnaires. Questionnaires and their identity information were saved separately and the numbers corresponding to identity information of the participants. All the researchers were required to ensure the confidentiality of identities of the participant. Researchers could not disclose their identity information to anyone without the permission of the participant. Identity information was kept in a locked filing cabinet, which can only be accessed by researchers. When necessary, members of government management departments or ethics committees could consult the identity information in the research unit according to regulations. The demographic characteristics of the participants are shown in Table 1. The results showed that two groups in all the indicators of general demographic data were comparable (*p* > 0.05) in Table 1.

**TABLE 1:** Characteristics of the sample.

Variable		Total sample (*n* = 809) *n* (*%*)	EFA sample (*n* = 404) *n* (*%*)	CFA sample (*n* = 405) *n* (*%*)	*t*/χ^2^/*Z*	*p*-value
Age, year	Mean (S.D.)	73.98(7.84)	73.76 (7.80)	74.20(7.89)	−0.802^a^	0.423
	Range	60∼95	60∼95	60∼93		
Gender	Male	489(60.44)	242(59.90)	247(60.99)	0.100^b^	0.752
	Female	320(39.55)	162(40.10)	158(39.01)		
Education level	Primary school or below	147(18.17)	78(19.31)	69(17.04)	−0.430^c^	0.667
	Junior high school	182(22.50)	86(21.29)	96(23.70)		
	Senior high school	226(27.94)	116(28.71)	110(27.16)		
	Junior college or above	254(31.40)	124(30.69)	130(32.10)		
Marital status	Married	707(87.39)	349(86.39)	358(88.40)	0.530^b^	0.467
	Other	102(12.61)	55(13.61)	47(11.60)		
Living status	Living alone	112(13.84)	62(15.35)	50(12.35)	2.430^b^	0.488
	Living with spouse only	537(66.38)	268(66.34)	269(66.42)		
	Living with children and spouse	127(15.70)	60(14.85)	67(16.54)		
	Living with children only	33(4.08)	14(3.47)	19(4.69)		
Monthly personal income (RMB)	<1000	38(4.70)	13(3.22)	25(6.17)	−0.262^c^	0.793
	1000∼2000	117(14.46)	62(15.35)	55(13.58)		
	2000∼4000	339(41.90)	188(46.53)	151(37.28)		
	≥4000	315(38.94)	141(34.90)	147(36.30)		
Physical condition	Very good	77(9.52)	40(9.90)	37(9.14)	−0.092^c^	0.927
	Good	264(32.63)	127(31.44)	137(33.83)		
	General	354(43.76)	183(45.30)	171(42.22)		
	Poor	82(10.14)	35(8.66)	47(11.60)		
	Very poor	32(3.96)	19(4.70)	13(3.21)		
Chronic illness	Yes	587(72.56)	291(72.03)	296(73.09)	0.113^b^	0.736
	No	222(27.44)	113(27.97)	109(26.91)		

*“^a^” represents the statistic is the t-value, “^b^” represents the statistic is the χ^2^-value, and “^c^” represents the statistic is the Z-value.*

### Data Analysis

The IBM SPSS Statistics version 26.0 (IBM Corporation, Armonk, NY, United States) was used for assessing the Chinese version of the BPNS (Jian et al., 2020) about item analysis, criterion validity, reliability analysis, and EFA. The IBM SPSS Amos version 24.0 was used for assessing its CFA and measurement invariance: (1) Item analysis: item analysis included the Pearson correlation analysis, assessing item-total correlations, and independent samples *t*-test, distinguishing the degree of discrimination of the items. The standard advice was to eliminate each item whose item-scale correlation was <0.30 (Polit and Beck, 2017). Scores were rank ordered and 27% of the highest and lowest scorers were selected to independent samples *t*-test, comparing statistical differences between two groups (McCowan and McCowan, 1999) for item discrimination; (2) Reliability: the Cronbach’s α coefficient of the total scale and subscales was calculated to examine internal consistency. The higher the value, the higher the reliability. Generally, the Cronbach’s α total coefficient, calculated to examine internal consistency, is considered acceptable if higher than 0.80 (Polit and Beck, 2017); and (3) Construct validity: EFA and CFA assessed construct validity. In terms of sample selection, two discrete samples were used to analyze EFA and CFA. EFA was used to assess the underlying construct of the items by using principal component analysis with Varimax rotation (Pett et al., 2003). Before conducting factor analysis, it was necessary to examine the applicability of the analysis. The *Kaiser–Meyer–Olkin* index (ranges from 0 to 1) greater than 0.50 and the result of the Bartlett’s test of sphericity were considered as eligible to perform EFA (Hill, 2011). The following criteria were used to determine the number of valuable factors: (a) eigenvalues greater than 1.0, (b) Cattell scree plot, (c) the percentage of total explained variance accounted for, and (d) items with loadings greater than 0.40 in absolute value (Pett et al., 2003). Then, CFA was performed to confirm the factorial structure identified in this exploratory study. The parameters in the model were estimated by maximum likelihood estimation. The parameters (Donna, 2009; Afthanorhan, 2013) used to test the goodness of fit of the model included the ratio of the χ^2^ value and the χ^2^/*df*, GFI, adjusted GFI (AGFI), CFI, Tucker–Lewis index (TLI), NFI, standardized root mean square residual (SRMR), and RMSEA. Model fit was deemed acceptable as the following values were met: χ^2^/*df* < 5, GFI > 0.90, AGFI > 0.90, CFI > 0.90, TLI > 0.90, NFI > 0.90, SRMR < 0.08, and RMSEA < 0.08 (Donna, 2009; Afthanorhan, 2013); (4) Criterion validity: criterion validity was evaluated by comparing the similarity of three dimensions and total scores of the Chinese version of the BPNS (Jian et al., 2020) and dimensions scores of the MHQ (Yuanjiang, 2003). The Pearson correlation analysis was used and a correlation coefficient ≤0.35 was considered low, from 0.36 to 0.67 was considered moderate, and 0.68 to 1.0 was considered high according to study by Taylor (1990); (5) Gender measurement invariance: multisample analysis was used to assess measurement invariance across sex and model fit parameters referred to CFA. With 0.05 as the test level, *p* < 0.05 indicated a statistically significant difference.

## Results

### Item Analysis

The Pearson correlation analysis assessed item-total correlations (Polit and Beck, 2017). Results showed that each item was positively correlated with the total score; item-total correlations ranged between 0.678 and 0.751 (*p* < 0.001), suggesting that each item was sufficiently homogeneous and need not to be eliminate. Factor correlation coefficients are given in Table 2.

**TABLE 2:** Item-total correlation (*n* = 809) and factor loadings (*n* = 404).

Dimension	English items	Chinese items	*R*	*F*_1_	*F*_2_	*F*_3_
Autonomy	1. That my choices were based on my true interests and values.	在我的生活中, 我的选择是基于我的真实的兴趣和价值观 兴趣和价值观	0.678*	–	0.836	–
	2. Free to do things my own way.	在我的生活中, 我自由地用自己的方式做事	0.706*	–	0.816	–
	3. That my choices expressed my “true self.”	在我的生活中, 我的选择表达了我的“真的自我”	0.701*	–	0.818	–
Competence	4. That I was successfully completing difficult tasks and projects.	在我的生活中，我曾经成功地完成困难的任务和计划	0.718*	–	–	0.801
	5. That I was taking on and mastering hard challenges.	在我的生活中，我曾经接受并且赢得困难的挑战	0.734*	–	–	0.858
	6. Very capable in what I did.	在我的生活中，我对于自己所做的事感到非常有能力	0.699*	–	–	0.718
Relatedness	7. A sense of contact with people who care for me, and whom I care for.	在我的生活中，我有一种联系着关心我的人们，以及我关心的人们的感觉	0.751*	0.821	–	–
	8. Close and connected with other people who are important to me.	在我的生活中，我感到亲密地联系着对于我重要的他人	0.720*	0.828	–	–
	9. A strong sense of intimacy with the people I spent time with.	在我的生活中，我对于身边的人们有一种强烈的亲密感	0.697*	0.825	–	–

“*”: p < 0.001; “–”: Factor loadings were lower than 0.60; F1 (Factor 1) = Relatedness; F2 (Factor 2) = Competence; F3 (Factor 3) = Autonomy.

The Chinese version of Jian et al. (2020) item discrimination of the BPNS was determined by testing the meaningfulness of the difference between the scale scores of the 27% upper and 27% subgroups after the raw scores were ranked from small to large (McCowan and McCowan, 1999). The independent samples *t*-test evaluated the difference between two groups for each item. Results showed that the statistical value of each item was statistically significant differences (*p* < 0.001) and that items had good discrimination. The results of the independent samples *t*-test are shown in Table 3.

**TABLE 3:** Independent samples *t*-test between two groups ($\bar{x}$ ±*s*).

Dimension	English items	Chinese items	High score group (*n* = 223)	Low score group (*n* = 235)	*t*-value	*p*-value
Autonomy	1. That my choices were based on my true interests and values.	在我的生活中，我的选择是基于我的真实的兴趣和价值观	6.57 ± 0.76	4.49 ± 1.07	−24.216	<0.001
	2. Free to do things my own way.	在我的生活中，我自由地用自己的方式做事	6.75 ± 0.56	4.69 ± 1.12	−25.142	<0.001
	3. That my choices expressed my “true self.”	在我的生活中，我的选择表达了我的“真的自我”	6.71 ± 0.60	4.52 ± 1.12	−26.303	<0.001
Competence	4. That I was successfully completing difficult tasks and projects.	在我的生活中，我曾经成功地完成困难的任务和计划	6.57 ± 0.72	4.13 ± 1.28	−25.317	<0.001
	5. That I was taking on and mastering hard challenges.	在我的生活中，我曾经接受并且赢得困难的挑战	6.52 ± 0.78	4.20 ± 1.16	−25.354	<0.001
	6. Very capable in what I did.	在我的生活中，我对于自己所做的事感到非常有能力	6.37 ± 0.90	4.08 ± 1.14	−23.875	<0.001
Relatedness	7. A sense of contact with people who care for me, and whom I care for.	在我的生活中，我有一种联系着关心我的人们，以及我关心的人们的感觉	6.77 ± 0.52	4.63 ± 1.09	−27.084	<0.001
	8. Close and connected with other people who are important to me.	在我的生活中，我感到亲密地联系着对于我重要的他人	6.75 ± 0.56	4.66 ± 1.19	−24.302	<0.001
	9. A strong sense of intimacy with the people I spent time with.	在我的生活中，我对于身边的人们有一种强烈的亲密感	6.67 ± 0.66	4.35 ± 1.12	−27.287	<0.001

### Reliability

The Cronbach’s α coefficient of the total scale was 0.877. The Cronbach’s α coefficient of three subscales were 0.826 (autonomy), 0.807 (competence), and 0.847 (relatedness).

### Construct Validity

#### Exploratory Factor Analysis

**FIGURE 1:**
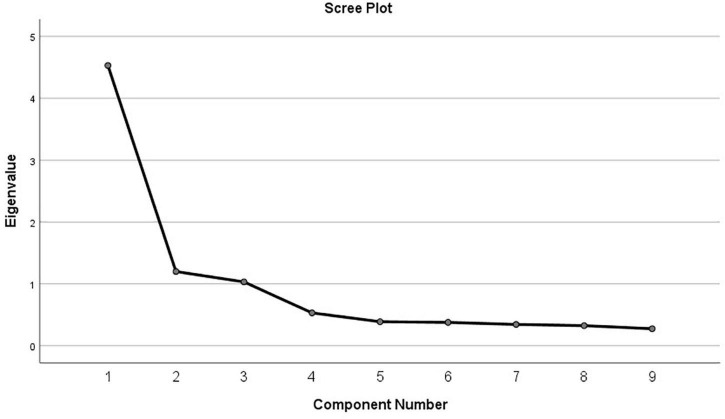
Cattell scree plot.

The *Kaiser–Meyer–Olkin* value obtained was 0.860 and the significance of the Bartlett’s test of sphericity was less than 0.001 (χ^2^ = 1723.111, *df* = 36). These results support the appropriateness of the factor analysis. Principal component analysis with Varimax rotation assessed the underlying construct of the items (Pett et al., 2003). Three factors were identified with eigenvalues greater than 1.0 and a total explained variance of 75.12% as shown in Table 4. All the factor loadings of the items were higher than 0.70. Note that the factor loadings of each item are shown in Table 2. Examination of the scree slope verified that a three-component solution was appropriate as shown in Figure 1.

**TABLE 4:** Explained variance of each factor (*n* = 404).

Component	Eigenvalue	Explained variance (%)	Cumulative explained variance (%)
1	4.531	50.34	50.34
2	1.199	13.32	63.67
3	1.031	11.46	75.12

#### Confirmatory Factor Analysis

**FIGURE 2:**
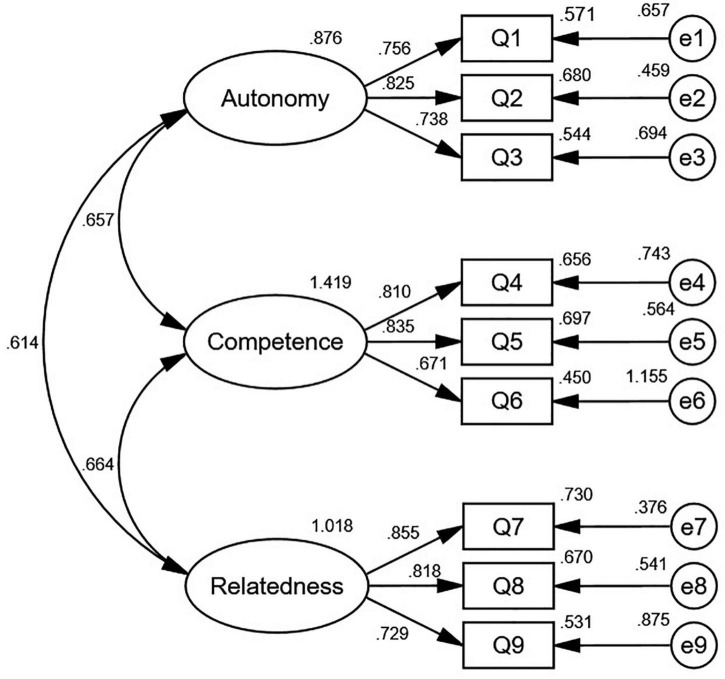
Confirmatory factor analysis standardized correlation diagram (*n* = 405).

Confirmatory factor analysis was performed using the IBM SPSS Amos version 24.0 (IBM Corporation, Armonk, NY, United States) to obtain a three-factor structural equation model. Results are shown in Table 5, indicating a good model fit. Figure 2 shows that the standardized factor loadings, ranging from 0.67 to 0.85, were statistically significant in the three-factor model.

**TABLE 5:** Model-fit index of confirmatory factor analysis (*n* = 405).

Inspected fit indices	Acceptable fit	Confirmatory factor analysis fit indices	Degree of fit
χ^2^/*df* (χ^2^ = 48.890, *df* = 24)	<5.0	2.037	Well
SRMR	<0.08	0.030	Well
RMSEA	<0.08	0.051	Well
GFI	>0.90	0.975	Well
AGFI	>0.90	0.952	Well
CFI	>0.90	0.985	Well
TLI	>0.90	0.978	Well
NFI	>0.90	0.972	Well

χ^2^/*df, the ratio of the χ^2^ value and the degrees of freedom; GFI, goodness of fit index; AGFI, adjusted GFI; CFI, comparative fit index; TLI, Tucker–Lewis index; NFI, normed fit index; SRMR, standardized root mean square residual; RMSEA, root mean square error of approximation.*

### Criterion Validity

The Pearson correlation analysis was used to assess the relationship between the Chinese version of the BPNS (Jian et al., 2020) and the MHQ (Yuanjiang, 2003). The three factors and total scores of the Chinese version of the BPNS (Jian et al., 2020) were significantly, moderately, and positively correlated with the happiness index of the MHQ (Yuanjiang, 2003) (*r* = 0.376, 0.394, 0.429, and 0.481; *p* < 0.001), indicating good criterion validity (Taylor, 1990). They also had a positive correlation with other positive dimensions of the MHQ (Yuanjiang, 2003) with statistically significant differences (*p* < 0.001) and a negative correlation with negative emotions with statistically significant differences (*p* < 0.001) correlation coefficient as shown in Table 6.

**TABLE 6:** The correlation coefficients between three dimensions and total scores of the Basic Psychological Need Satisfaction Scale (BPNS) and dimensions of the Multiple Happiness Questionnaire (MHQ) (*r*) (*n* = 507).

Criterion	Life satisfaction	Positive affect	Negative affect	Subjective vitality	Self-worth	Health concern	Positive relation	Altruism commitment	Personal growth	Happiness index
Autonomy	0.549	0.408	–0.150	0.489	0.520	0.452	0.344	0.369	0.628	0.376
Competence	0.518	0.457	–0.150	0.535	0.571	0.493	0.351	0.488	0.575	0.394
Relatedness	0.554	0.461	–0.243	0.441	0.476	0.447	0.535	0.415	0.533	0.429
Total score	0.650	0.533	–0.217	0.590	0.631	0.560	0.491	0.513	0.697	0.481

*All the above correlation coefficients are p < 0.001.*

### Gender Measurement Invariance

Since the two baseline models for each gender were the same, multi-sample analysis was then conducted. First, multi-sample analysis with the unconstrained model (model 1) showed an acceptable baseline model for both males and females [χ^2^ (48) = 74.620, *p* < 0.05, CFI = 0.984, TLI = 0.976, and RMSEA = 0.037]. Then, to test the invariance of the factor loadings across gender, factor loadings were constrained to be equal across two groups. Multi-sample analysis revealed that model 2 was acceptable [χ^2^ (54) = 80.770, *p* < 0.05, CFI = 0.984, TLI = 0.979, and RMSEA = 0.035]. Also, the χ^2^ difference test between baseline model and constrained model was not significant [χ^2^ (6) = 6.150, *p* = 0.407 > 0.05, ΔCFI = 0], suggesting that factor loadings of both the gender groups were invariant. In addition to the factor loadings, factor variances and covariances were also constrained to be equal across two groups. Multi-sample analysis showed that model 3 was acceptable [χ^2^ (60) = 92.317, *p* < 0.05, CFI = 0.981, TLI = 0.977, and RMSEA = 0.037]. Moreover, the χ^2^ difference test between two constrained models was not significant [χ^2^ (6) = 11.548, *p* = 0.073 > 0.05, ΔCFI = 0.003]. It suggested that, aside from the factor loadings, factor variances and covariances were also invariant across gender. Finally, besides the constraints mentioned above, residuals were also constrained to be equal across two groups. Multi-sample analysis revealed that model 4 was acceptable [χ^2^ (69) = 106.600, *p* < 0.05, CFI = 0.977, TLI = 0.977, and RMSEA = 0.037]. Additionally, the χ^2^ difference test between two constrained models was not significant [χ^2^ (9) = 14.283, *p* = 0.113 > 0.05, ΔCFI = 0.004]. Therefore, all these results revealed that the factor loadings, factor variances and covariances, and residuals were invariant across gender, as shown in Table 7. Item covariance matrices of each gender group are shown in Table 8.

**TABLE 7:** Goodness-of-fit index for the cross gender measurement invariance models (*n* = 405).

Models	χ^2^/*df*	*p*-value	RMSEA (90%CI)	CFI	TLI	Δχ^2^	Δ*df*	*p*-value	ΔCFI
Model1	1.555(74.620/48)	0.008	0.037(0.019∼0.053)	0.984	0.976				
Model2	1.496(80.770/54)	0.011	0.035(0.017∼0.050)	0.984	0.979	6.150	6	0.407	0.000
Model3	1.539(92.317/60)	0.005	0.037(0.021∼0.051)	0.981	0.977	11.548	6	0.073	0.003
Model4	1.545(106.600/69)	0.002	0.037(0.022∼0.050)	0.977	0.977	14.283	9	0.113	0.004

**TABLE 8:** Item covariance matrices of each gender group (*n* = 405).

	*Item*1	Item2	Item3	Item4	Item5	Item6	Item7	Item8	Item9
**Male (*n* = 247)**									
Item1	1.568								
Item2	1.051	1.560							
Item3	0.915	0.969	1.642						
Item4	0.750	0.860	0.848	2.344					
Item5	0.723	0.755	0.670	1.552	1.941				
Item6	0.534	0.518	0.814	1.267	1.109	1.992			
Item7	0.518	0.637	0.535	0.827	0.905	0.721	1.453		
Item8	0.553	0.658	0.596	0.808	0.845	0.709	1.093	1.781	
Item9	0.645	0.606	0.551	0.811	0.763	0.664	0.992	1.152	1.892
**Female (*n* = 158)**									
Item1	1.477								
Item2	0.778	1.189							
Item3	0.662	0.725	1.262						
Item4	0.787	0.553	0.576	1.784					
Item5	0.629	0.516	0.552	1.042	1.693				
Item6	0.734	0.634	0.773	0.900	0.959	2.212			
Item7	0.590	0.540	0.641	0.525	0.708	0.709	1.224		
Item8	0.604	0.462	0.550	0.523	0.630	0.685	0.912	1.335	
Item9	0.540	0.450	0.612	0.471	0.613	0.635	0.912	0.885	1.764

### Comparison of Three Basic Psychological Needs Satisfaction of Older Adults and College Students

Comparing the total score and three subscales scores of basic psychological needs satisfaction between older adults and college students (Jian et al., 2020) is shown in Table 9. The independent samples *t*-test showed that two groups had statistically significant differences in the scores of three dimensions and the total scale (*p* < 0.001). The three basic psychological and overall needs of older adults were satisfied. These levels were higher than those of college students.

**TABLE 9:** Basic psychological needs satisfaction of older adults and college students ($\bar{x}$ ± *s*).

Dimensions	Older adults (*n* = 809)	College students (*n* = 291)	*t*-value	*p*-value
Autonomy	5.62 ± 1.06	4.72 ± 1.30	11.669	<0.001
Competence	5.34 ± 1.17	4.92 ± 1.17	5.252	<0.001
Relatedness	5.71 ± 1.10	4.85 ± 1.19	11.189	<0.001
Total scale	5.56 ± 0.92	4.83 ± 0.96	11.474	<0.001

## Discussion and Conclusion

### Reliability and Validity of the Chinese Translation of the Basic Psychological Need Satisfaction Scale Based on Self-Determination Theory

In this study, the simplified Chinese version of the BPNS (Jian et al., 2020) is sufficiently homogeneous and item discrimination and presented satisfactory reliability, construct validity, and criterion validity for use among Chinese older adults in Mainland China. First, according to the result of item analysis, nine items of the scale are wholly saved in this study, which is the same as Jian et al. (2020). Second, it demonstrated that the scale has satisfactory internal consistency according to study by Polit and Beck (2017). The total Cronbach’s α coefficient of the Chinese translation of the BPNS (Jian et al., 2020) in older adults was 0.877 and three subscales were 0.826 (autonomy), 0.807 (competence), and 0.847 (relatedness). The Cronbach’s α coefficients of the total scale and three subscales in this study are equivalent to college students (Jian et al., 2020), both good internal consistency. Third, principal component analysis showed that the scale has a clear structure and that the attribution of each item is consistent with that reported by Jian et al. (2020). Each item factor loading was above 0.70 and the cumulative contribution rate of the three principal components was 75.12%. Because each dimension is clear and explanatory, the scale has good construct validity. The model fits the observed data well and supports EFA, which also verifies the construct validity. Finally, criterion validity is testified from three aspects of positive mental health, passive mental health, and happiness index, indicating that the scale has good criterion validity and the relationship between basic psychological needs satisfaction and well-being is supported. In a word, the internal construct of the scale was relatively stable when tested with older participants. Meanwhile, external validity is good.

### Comparison of the Three Basic Psychological Needs Satisfaction of Older Adults and College Students

With increasing age, people tend to value intrinsic goals more than extrinsic goals (Behzadnia et al., 2020). Compared with young people who value extrinsic goals such as money and fame, older adults who pursue intrinsic goals report higher satisfaction with their three basic psychological needs. This study showed that the satisfaction levels of the three basic psychological needs and the overall needs of older adults were higher than those of college students; the differences were statistically significant (*p* < 0.001). Compared to interactions with colleagues and acquaintances, interacting with family members and friends usually inspires feelings of relatedness and autonomy (Ryan and Deci, 2017). In this study, except empty-nest older adults living alone (13.84%), without spouses, children, or other family members in their households, most participants cared for their spouses or the family. Compared with college students, older adults expressed higher overall intrinsic satisfaction. Higher individual levels of subjective socioeconomic status and household income predict higher need-fulfillment levels (Di Domenico and Fournier, 2014). In this study, 80.84% of older adults had a monthly income of more than 2,000 Yuan; older adults living as a couple or with children had higher economic levels. Compared with college students who are likely to have no income, older adults generally have an advantage at the economic level. Satisfaction with autonomy, competence, relatedness, and overall needs was higher.

### Feasibility of the Chinese Translation of the Basic Psychological Need Satisfaction Scale Based on Self-Determination Theory With Older Adults

There are multiple versions of the BPNS at home and abroad, but those used to assess older adults in China have been lacking. This study revealed that the Chinese version of the BPNS (Jian et al., 2020) was factor invariant across gender in a sample of older adults. This study adopted the Chinese version of the BPNS (Jian et al., 2020) based on contrast various versions of the basic psychological needs scale domestic and overseas and overall characteristics of the older adults. Results showed that the scale had good reliability and validity with senior adults. What is more, the time required to complete the Chinese version of the BPNS (Jian et al., 2020) was 5–10 min per person during the data collection stage. Therefore, the data collection process of the Chinese version of the BPNS (Jian et al., 2020) is relatively smooth because it contains only nine items, is simple, and is easy to understand. This study found that many older adults could complete the Chinese version of the BPNS (Jian et al., 2020) independently, indicating that it was an appropriate measure to test satisfaction with basic psychological needs. In future, the scale could help community staff to assess satisfaction with basic psychological needs of the senior adults rapidly to enact timely interventions to promote healthy aging.

### Limitations

This study had some limitations. Firstly, it did not investigate groups of older adults with varying characteristics. Future researchers could consider conducting based on these differences such as illness or not. Secondly, Fuzhou is a coastal city within certain regional and urban–rural differences reflected in the psychological needs of older adults of that region. Future study could adjust the sample coverage to reflect wider characteristics and a more diverse range of older adults in China to assess satisfaction with basic psychological needs. In addition, although the aim of this study was to examine the application of the BPNS with elders in a Chinese context, this study did not provide evidence across various cultures or languages within mainland in China neither it provide evidence for the equivalence of the BPNS between China and the western cultures. Future study is encouraged to explore the BPNS across cultures and/or languages within China and between China and the west to examine the equivalence of the BPNS and enhance cross-cultural comparison and application of self-determination theory.

## Data Availability Statement

The original contributions presented in the study are included in the article/supplementary material, further inquiries can be directed to the corresponding author.

## Ethics Statement

This study involving human participants was reviewed and approved by the Ethics Review Committee of Fujian Medical University. The participants signed their written informed consent to participate in this study.

## Author Contributions

YuY was responsible for recruitment, data collection, analysis and interpretation of the data, and drafting the manuscript. MY, YoY, and QY was responsible for recruitment, data collection, and critical review of the manuscript. TL was responsible for the study conception and design, supervision of the study, provision of administrative and material support, analysis and interpretation of the data, and drafting and critical review of the manuscript. All authors have contributed significantly and in agreement with the content of the manuscript.

## Conflict of Interest

The authors declare that the research was conducted in the absence of any commercial or financial relationships that could be construed as a potential conflict of interest.

## Publisher’s Note

All claims expressed in this article are solely those of the authors and do not necessarily represent those of their affiliated organizations, or those of the publisher, the editors and the reviewers. Any product that may be evaluated in this article, or claim that may be made by its manufacturer, is not guaranteed or endorsed by the publisher.

## Footnotes

1
https://www.randomizer.org/
